# Research progress of confocal laser endomicroscopy in malignant respiratory diseases: A review

**DOI:** 10.1097/MD.0000000000048639

**Published:** 2026-08-14

**Authors:** Fan Zhiliang, Li Qian, Tang Yilian, Pu Xiang, Chai Yihui

**Affiliations:** a Department of Basic Medicine, Guizhou University of Traditional Chinese Medicine, Guizhou, China.

**Keywords:** confocal laser endomicroscopy, lung cancer, malignant respiratory diseases

## Abstract

The diagnosis and monitoring of malignant respiratory diseases are heavily reliant on imaging techniques and tissue biopsies. Confocal laser endomicroscopy (CLE) is an advanced real-time imaging modality with high resolution, capable of visualizing the morphology and structure of pulmonary tissue in conjunction with conventional bronchoscopy. Within the realm of malignant respiratory diseases, it enables differentiation between normal and abnormal regions in the central airways, lung parenchyma, mediastinal lymph nodes, and pleura. Although CLE has not yet been integrated into routine clinical practice, it has the potential to guide biopsies, thereby enhancing diagnostic yield while mitigating the occurrence of associated complications. Moreover, this technology holds significant potential for elucidating disease pathogenesis, facilitating intraoperative assessment, and monitoring therapeutic efficacy. Looking towards the future, the integration of CLE with artificial intelligence represents a promising direction for the development of this technology. This review encapsulates the latest advancements of CLE in the context of malignant respiratory diseases, with a particular emphasis on its application in the diagnosis and treatment of lung cancer.

## 1. Introduction

The diagnosis of malignancies in respiratory diseases predominantly depends on imaging methodologies like chest CT scans and tissue biopsies, both of which possess inherent drawbacks. Imaging techniques such as high resolution computed tomography, are constrained by their spatial resolution and their inability to provide real-time feedback on lesions, in addition to exposing patients to sustained ionizing radiation exposure.^[1]^ Tissue biopsies, including those performed minimally invasively via bronchoscopy, are plagued by false-negative results, diminished diagnostic rates due to inconsistent biopsy sites, and unavoidable complications.^[2]^

Emerging as a novel “optical biopsy” technique, confocal laser endomicroscopy (CLE) integrates ex vivo histological examination with in vivo imaging, thus facilitating imaging of tissue structure and morphology at a cellular level. This provides clinicians with real-time feedback, thereby enabling accurate diagnosis and treatment planning.^[3]^ Although still in its infancy, several studies hint at the potential of CLE in the diagnosis and management of malignant respiratory diseases. This paper aims to review research based on CLE and discusses its applications in lung cancer, mediastinal lymph nodes, and pleural lesions, with a focus on specific anatomical sites (Table 1).

**Table 1:** Clinical study of CLE in malignant diseases of respiratory system.

Disease type	Imaging site	pCLE	nCLE	Potential application value
Lung cancer	Air passage	Detection of intrabronchial tumors and nonneoplastic lesions^[4–6]^	–	- Improve early detection of lung cancer - Guiding tools for transbronchoscopy and percutaneous transthoracic biopsy - Improve the diagnostic rate of transbronchoscopy and percutaneous needle aspiration biopsy
	Pulmonary interstitium	Identification of malignant nodules^[7–15]^	Detection of peripheral pulmonary nodules^[16–22]^	
Metastatic lymph	Mediastinal lymph nodes	–	Identification of malignant cells in lymph nodes^[18,23]^	- Distinguish between malignant, granulomatous and reactive lymph nodes - Improve the accuracy of lung cancer staging
Pleuropathy	Pleura	- Distinction between normal and abnormal pleura^[24,25]^ - Detection of malignant cells in pleural effusion^[26]^ - Detection of visceral pleural invasion^[27]^	Differentiation of pleural fibrosis from malignant mesothelioma^[25]^	- Guidance tool for pleural biopsy - Differentiation of benign and malignant lesions - Intraoperative evaluation

nCLE = needle confocal laser endomicroscopy, pCLE = probe-based confocal laser endomicroscopy.

### 1.1. Technical background of CLE

Indeed, CLE, also recognized as fibred confocal fluorescent microscopy, it can be utilized in conjunction with any endoscope and complementary tools. It can also be used alongside specialized endoscopic techniques such as narrow band imaging and biolayer interferometry. These combinations allow for real-time, high-resolution imaging of target areas. The target areas can include alveoli, pleura, and lymph nodes. This capacity for detailed, real-time imaging greatly enhances the potential for accurate diagnosis and effective treatment planning.

The principle of CLE involves the guidance of flexible miniature probes through the working channel of an endoscope to the target area. Here, a low-power laser, typically with a wavelength of 488 nm, irradiates the tissue under examination. Reflected light from the tissue then traverses back through the detection pinhole to the detector. Concurrently, light emanating from outside the focal point is obstructed by the pinhole, which prevents it from interfering with image formation. As a result, a confocal image is generated. Imaging is implemented one focal point at a time, and a computer-controlled point-by-point scanning process is employed to generate two-dimensional or three-dimensional images of the specimen.^[28]^

Contemporary clinical studies have testified to the utility and safety of CLE in gastrointestinal and pancreaticobiliary diseases.^[29]^ In 2007, Thiberville^[4]^ first introduced this technology into the respiratory system and established that CLE imaging primarily depends on the elastic protein components. This discovery paved the way for further exploration of the clinical value of CLE. Presently, a commercial CLE system (cellvizio; Mauna Kea Technologies, Paris, France) is available for respiratory diseases. It enables a field of view up to 600 µm, a confocal depth ranging from 0 to 50 µm, and a lateral resolution reaching 3.5 µm, thereby providing real-time imaging at speeds of 9 to 12 frames per second.

Currently, CLE has 2 primary clinical applications: probe-based confocal laser endomicroscopy (pCLE) and needle confocal laser endomicroscopy (nCLE). While pCLE is more commonly used in clinical practice, nCLE, being equipped with probes of smaller diameters, holds the potential to image different anatomical structures, including individual malignant cells and lymph nodes. This makes nCLE particularly suitable for the assessment of lymph nodes. Such advancements in technology are paving the way for improved diagnosis and treatment planning in the field of respiratory diseases.

## 2. Clinical application

### 2.1. Lung cancer

Lung cancer is indeed the most prevalent malignant tumor globally and the leading cause of cancer-related deaths in males.^[30]^ While tissue biopsy continues to be the gold standard for the accurate diagnosis of lung cancer, it’s important to consider the associated complications that may occur during biopsy procedures. CLE, serving as a minimally invasive “optical biopsy” tool, offers real-time histopathological information of the target region. This promises to overcome the limitations associated with traditional biopsy methods, making it a potentially valuable tool in the diagnosis and management of lung cancer.

#### 2.1.1. Central lung cancer

Thiberville^[4]^ indeed first reported the clinical application of pCLE in preinvasive lesions of the airways. They observed that pCLE images of precancerous lesions show disordered fiber networks, which is distinct from normal airways. Later in 2015, Wellikoff^[5]^ found speckled elastic fibers, disorganized fiber arrangements, and fragmentation in pCLE imaging of non-small cell lung cancer patients, which correlated with histopathological findings. Luo^[7]^ reported domestically for the first time on pCLE detection of different sites of lung cancer lesions, revealing that 11 out of 14 new lesions lacked visible fiber structures, thus failing to identify malignant lesions. Similarly, Seth^[8]^ found no significant advantage of pCLE imaging in distinguishing between benign and malignant nodules, possibly due to the absence of contrast agents. Previous studies have indeed confirmed the role of fluorescein in characterizing epithelial cells.^[31]^ However, because fluorescein is unable to penetrate the bronchial mucosal epithelial layer for cell staining, its application in lung cancer diagnosis is limited.^[32]^

**Figure 1.**
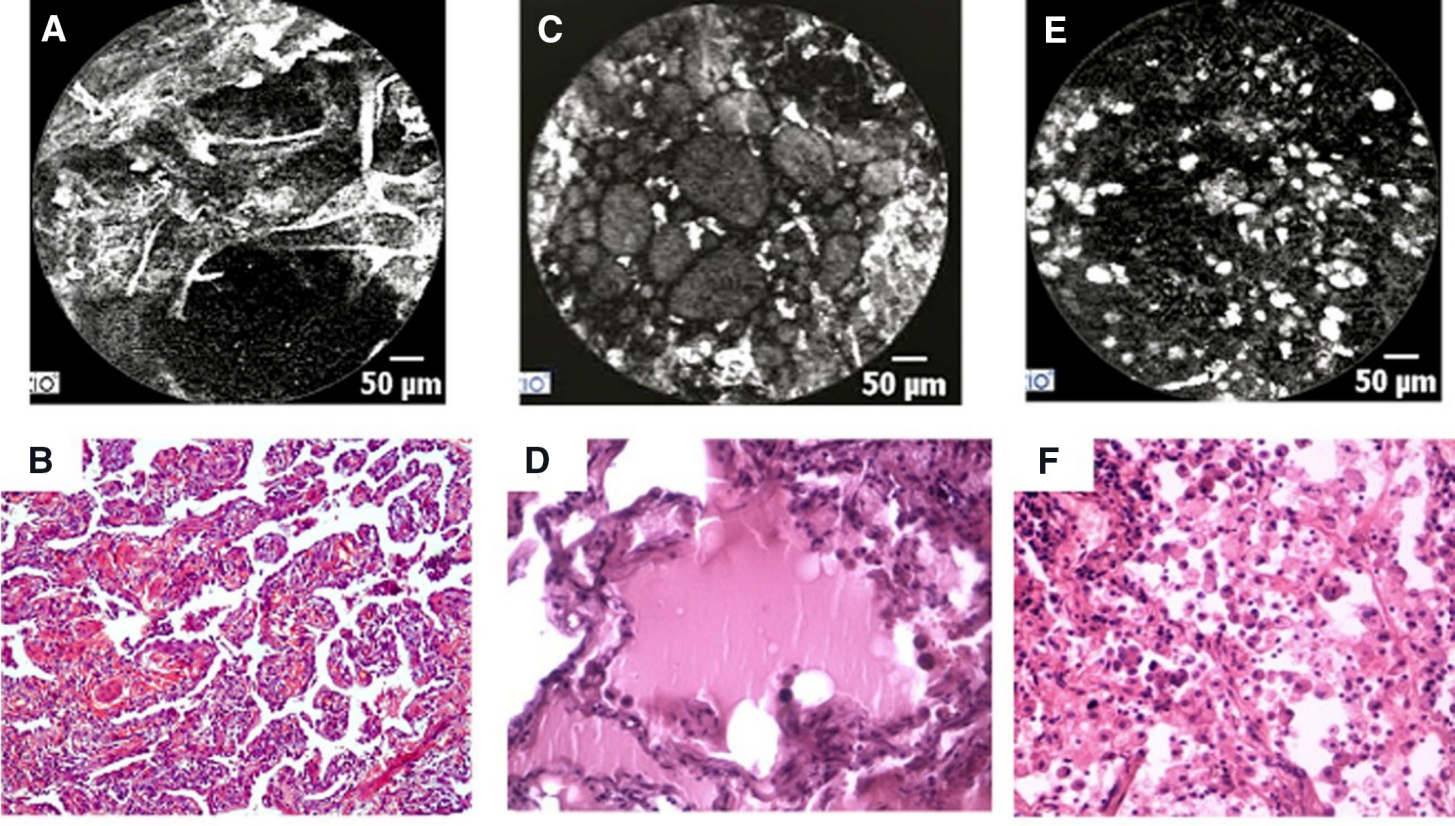
Characteristics of lung cancer. pCLE images: (A) thickened alveolar wall and increased alveolar space; (C) alveolar edema; (E) highly luminous macrophage masses; hematoxylin–eosin staining X200 times: (B) thickened alveolar wall with lymphocyte infiltration; (D) alveolar edema, with eosinophilic alveolar secretions; (F) massive alveolar macrophage infiltration around lung cancer tissue.^[11]^ pCLE = probe-based confocal laser endomicroscopy. Reproduced with permission from Sorokina et al^[11]^ © John Wiley and Sons.

**Figure 2.**
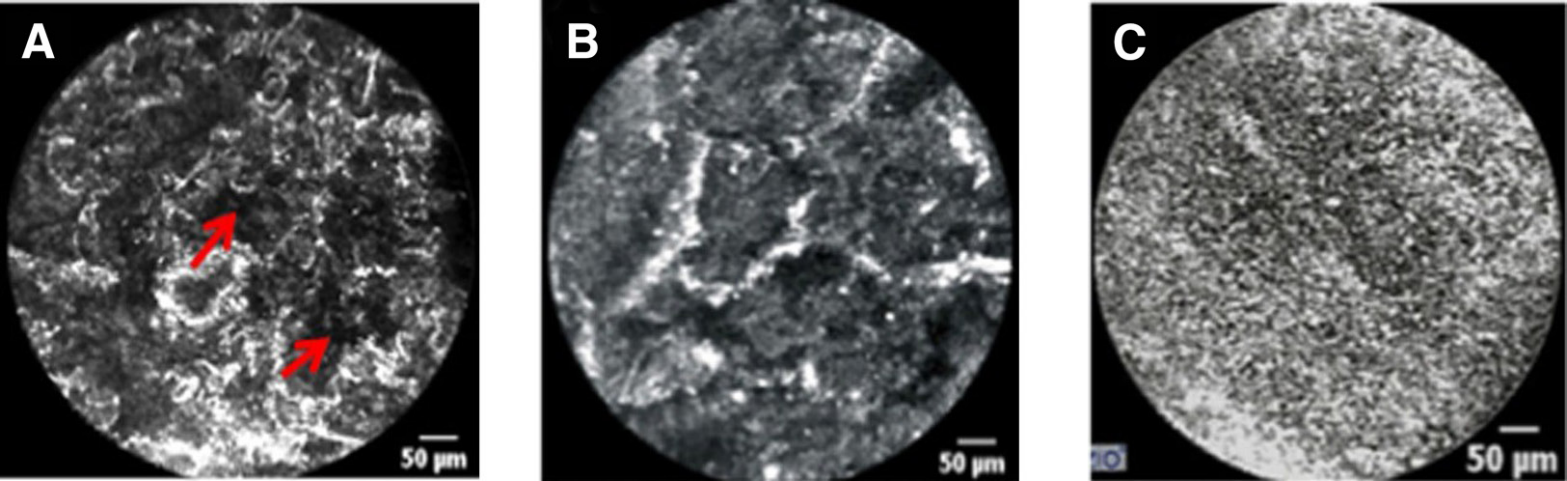
pCLE image characteristics of different types of lung cancer. (A) Lung adenocarcinoma: interstitial hyperfluorescence penetration (red arrow), “black hole sign”; (B) Lung squamous cell carcinoma: amorphous mass (“ twin’’ branches with high fluorescent fibers); (C) small cell lung cancer: unstructured mass, visible light scattering effect.^[11]^ pCLE = probe-based confocal laser endomicroscopy. Reproduced with permission from Sorokina et al^[11]^ © John Wiley and Sons.

Given the limitations of fluorescein, researchers have indeed started exploring new fluorescent contrast agents. In this context, a study conducted pCLE examinations on 32 patients suspected of malignant tumors after spraying acriflavine, and observed no related complications during the examination.^[6]^ Subsequently, a prospective comparison of 75,522 pCLE images from 56 different sites was made with histopathological results. The sensitivities, specificities, and accuracies of pCLE in diagnosing malignant tumors were found to be 96.0%, 87.1%, and 91.0%, respectively. Recent studies^[9]^ have also confirmed that acriflavine provides additional diagnostic information, thereby improving the accuracy of pCLE in diagnosing malignant tumors. Additionally, methylene blue, which needs to be used in conjunction with the 660 nm Cellvizio system, has been shown to have a similar diagnostic efficacy as acriflavine.^[10]^ Sorokina^[11]^ stained postoperative lung cancer nodules with methylene blue and conducted pCLE examinations. They discovered 3 characteristic features specific to lung cancer: thickening of alveolar walls leading to alveolar atrophy, alveolar edema, and abundant macrophages. These findings highlight the potential of these new contrast agents in improving the diagnostic accuracy of pCLE in lung cancer (Fig. 1). In lung adenocarcinoma, pCLE images show a specific characteristic often referred to as the “black hole sign.” This is described as high fluorescence permeation in the interstitium, which means there is significant uptake and distribution of the fluorescent dye. This unique pattern helps in the identification and differentiation of lung adenocarcinoma from other types of lung cancer (Fig. 2A); lung squamous cell carcinoma indeed presents itself uniquely in pCLE images. It appears as amorphous masses, often described as “twin” branching high-fluorescence fibers. This imagery helps in its identification and differentiation from other types of lung cancer (Fig. 2B); in the case of small cell lung cancer, it’s characterized in pCLE imaging as unstructured masses with visible light scattering effects. This unique imaging pattern helps in differentiating small cell lung cancer from other types of lung cancer (Fig. 2C). Some studies have indicated that methylene blue offers higher safety than acriflavine.^[33]^ It should be emphasized that, in addition to contrast agents, molecular targeted agents also play a significant role in the identification of malignant tumors.^[34,35]^ However, their safety and efficacy necessitate rigorous further validation.

Beyond the capability of differentiating between benign and malignant lesions, pCLE offers the additional benefits of performing intraoperative assessments and providing real-time monitoring of treatment responses. This aligns with findings from pediatric surgical oncology, where ex vivo FCM allowed immediate resection margin expansion in a case of atypical Spitz nevus, avoiding second surgeries.^[36]^ A notable application of this was developed by Guisier,^[12]^ who utilized a real-time quantitative imaging system based on pCLE and NucView 488 Caspase 3 substrate, a fluorescent probe targeting activated Caspase 3, in order to evaluate the sensitivity of EGFR inhibitors. They proceeded with injecting erlotinib-resistant, insensitive, and hypersensitive cell lines into mice and subsequently performed imaging on both in vivo and ex vivo tumor xenografts. The assessment of apoptosis revealed significant differences among the 3 cell lines. Indeed, a recent case study by Terra^[37]^ showcased the potential of pCLE in enhancing the precision of tumor identification during surgical interventions. In this particular case of a young male diagnosed with bronchial carcinoid, pCLE was employed during a robot-assisted bronchoplasty. This allowed for the accurate pinpointing of the tumor area, subsequently circumventing the need for lung resection. Therefore, the integration of pCLE imaging technology with existing clinical practices could potentially signify a superior approach in the treatment of airway tumors, offering improved precision and potentially reducing the need for more invasive procedures.

#### 2.1.2. Peripheral lung cancer

Indeed, with the increasing prevalence of lung cancer screening programs and the widespread use of chest CT scans, there is a surging incidence of detected solitary pulmonary nodules (SPN), particularly peripheral lung nodules, which poses a significant diagnostic challenge for interventional pulmonologists.^[38]^ At present, numerous innovative techniques such as radial endobronchial ultrasound (r-EBUS), virtual navigation, and cone-beam CT are employed in clinical practice for the diagnosis of peripheral lung cancer. However, each of these methodologies has its own set of limitations.^[13]^ The integration of CLE with these existing techniques could potentially represent a novel and improved approach to this diagnostic quandary.

Arenberg^[14]^ introduced the first set of criteria for diagnosing peripheral lung nodules using pCLE, which is based on the “dense alveolar structure” also known as the Columbus grading system. When these criteria were applied for evaluation, the sensitivity for detecting lung cancer increased to 70% to 80% and specificity ranged between 58% to 74%. Further, in the study by Luo,^[7]^ the pCLE imaging features of peripheral lung lesions included “circinate” structural destruction, compressed alveoli, clustered fibers, thickened alveolar walls, and black hole signs. However, due to the limited flexibility of the pCLE probe tip, the exploration of the upper lobes of both lungs, which requires a higher degree of curvature, was not conducted in this study. In 2015, Hassan^[15]^ demonstrated the potential of pCLE in aiding the visualization of the biopsy site for accurate localization of SPN during biopsy procedures conducted with r-EBUS or virtual navigation. They even managed to reach the upper lobes of both lungs using a more flexible confocal probe (cholangioflex) with an outer diameter of only 0.6 mm. In an effort to further validate these findings, the research team included 48 patients with malignant SPNs and found a diagnostic accuracy of 79.2% for r-EBUS combined with pCLE. However, when the cholangioflex probe was used, despite its ability to probe all SPNs, it was found that both spatial resolution and image quality were compromised.^[39]^

The introduction of miniaturized probes in nCLE might indeed present an innovative solution. Past studies on nCLE have largely been limited to pancreatic diseases and abdominal lymph nodes.^[16]^ However, Wijmans^[17]^ were the first to apply nCLE in patients with peripheral lung nodules, where they observed nCLE image features of lung adenocarcinoma as dark aggregates within alveolar spaces. Su^[18]^ further characterized the nCLE features of adenocarcinoma and tuberculosis. In adenocarcinoma, they found a disordered arrangement of fibrous tissue accompanied by spot or black hole structures, while in tuberculosis, they observed matrix destruction and partial tissue structural disorder with uniform density.In the study conducted by Wijmans,^[19]^ 22 patients with suspected or confirmed lung cancer were evaluated using nCLE before undergoing endoscopic ultrasound-guided fine-needle aspiration. No adverse reactions were reported. The researchers identified 3 nCLE image features associated with lung cancer: enlarged pleomorphic cells, dark clumps, and directed flow. These features helped achieve a 90% accuracy rate in lung cancer diagnosis with high interobserver and intraobserver consistency. Following this, Kramer^[20]^ established 4 nCLE criteria for airways and lung parenchyma (elastic fiber bundles and static images representing tracheal, bronchial epithelium, and alveoli). These criteria distinguished malignant nCLE features with a 95% accuracy rate. Moreover, the combination of nCLE with robot-assisted navigation bronchoscopy has been shown to optimize the analysis of peripheral lung nodules, thereby enhancing diagnostic rates.^[21,40]^ Given that nCLE operates based on the principle of reverse contrast imaging, it fails to offer specific detection of individual tumor cells. Furthermore, the interpretation of nCLE images heavily relies on experienced observers.^[19,20,22]^ In response to this, Kennedy^[23]^ proposed a novel approach that combines near-infrared (NIR) detection technology with nCLE. This approach uses a cancer-targeting tracer (paflonacianine) to achieve real-time detection at the cellular level. Their study conducted NIR-nCLE imaging on specimens from 20 patients with SPN after resection. The images were evaluated by 5 nonexpert observers, and the results showed a diagnostic accuracy of 97% for malignant nodules. The interobserver consistency was also excellent (κ = 0.95). Moreover, this study found that even ground-glass opacities, which are less dense and more challenging to identify, can be rapidly, accurately, and consistently detected using NIR-nCLE.

### 2.2. Mediastinal lymph nodes

**Figure 3.**
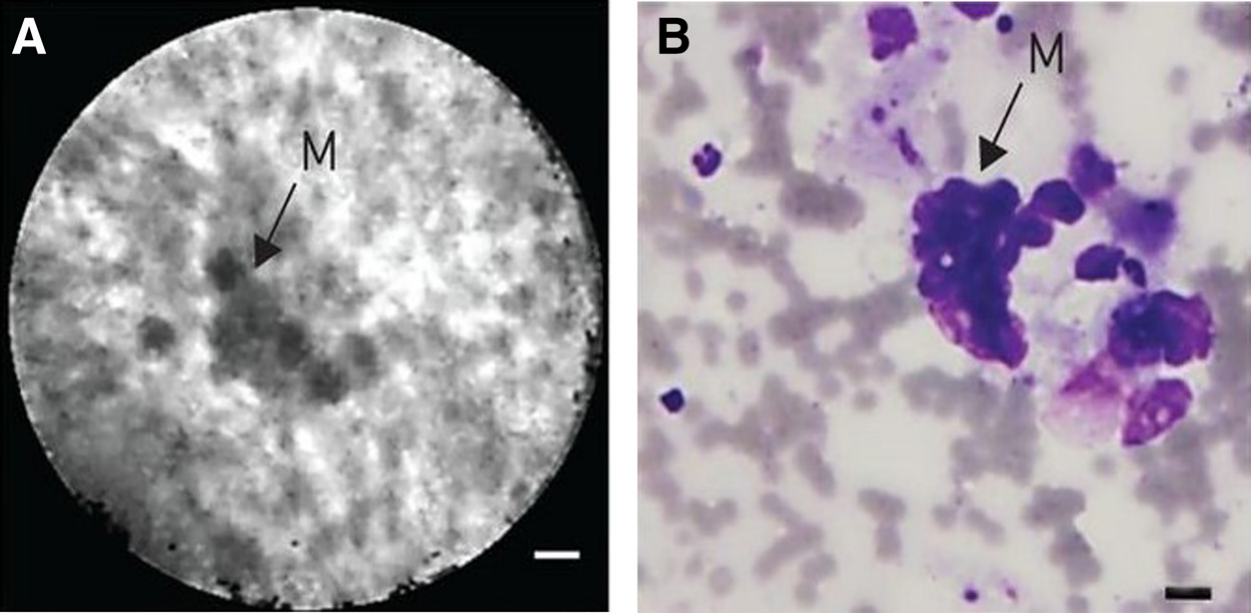
Characteristics of metastatic lymph nodes. (A) nCLE images: dark pleomorphic cells and dark masses (M); (B) fine needle aspiration cytology: a cluster of malignant cells (M) (Wijmans et al).^[19,26]^ nCLE = needle confocal laser endomicroscopy. Reproduced with permission from Wijmans et al^[19]^ © ERS 2026.

Benias^[41]^ evaluated the safety and effectiveness of nCLE in staging 28 patients with malignant tumors or lymphadenopathy using endoscopic ultrasound. The procedure was carried out successfully in all patients without any associated complications. The researchers were able to diagnose 17 patients with malignant tumors, 4 with malignant lymphoma, and 7 with benign lymph node hyperplasia based on the different features observed in the nCLE images. Particularly in malignant lymph nodes, dark pleomorphic cells were easily identifiable. Furthermore, Wijmans^[19]^ studied 21 mediastinal lymph nodes using nCLE and identified unique features of malignant lymph nodes, including dark pleomorphic cells and dark clumps (Fig. 3). These findings were consistent with nCLE descriptions in pancreatic cancer.^[24]^ When these criteria were applied, the diagnostic accuracy for metastatic lymph nodes reached 89%.Therefore, nCLE demonstrates unique advantages in the evaluation of lymph nodes.

### 2.3. Pleura

Indeed, both pCLE and nCLE have been leveraged to observe pleural diseases through endoscopic channels. They offer real-time visualization of pleural lesions, help to guide targeted pleural biopsies, and assess the severity of pleural fibrosis. For instance, Bonhomme^[25]^ evaluated 3 referred patients undergoing thoracoscopy using pCLE. They were able to accurately distinguish normal pleura, malignant pleuritis, and malignant mesothelioma based on the characteristics of the pCLE images. Moreover, a multicenter prospective study^[26]^ that distinguished benign from malignant pleural lesions using pCLE or nCLE underscored the role of CLE in guiding pleural biopsy. Fifteen patients suspected of having malignant mesothelioma underwent CLE examination before pleural biopsy. The procedure successfully distinguished abnormal pleura from pleural fibrosis. These studies illustrate the potential of pCLE and nCLE in diagnosing and managing pleural diseases.

The use of pCLE has proven to be valuable in guiding pleural biopsy, enhancing diagnostic accuracy, reducing the need for repeat biopsy procedures, and avoiding related adverse events. It has also been effective in diagnosing malignant pleural effusion. In a preliminary study,^[27]^ 100 patients with pleural effusion underwent thoracentesis, which was followed by a pCLE examination of the effusion stained with toluidine blue and acriflavine hydrochloride. The sensitivity of these 2 stains in detecting malignant effusion was found to be 81% and 87%, respectively, with specificities of 92% and 99%, respectively. Sawada^[42]^ also evaluated the feasibility of using intraoperative pCLE to detect visceral pleural invasion in lung cancer patients. They found an accuracy rate of 86.7% in diagnosing visceral pleural invasion with pCLE. However, it’s important to note that these are initial findings and larger prospective studies are needed to confirm these results.

## 3. Conclusion

Absolutely, CLE is a noninvasive, nontraumatic, high-resolution microscopic imaging technology. It’s been proven to be safe and feasible for use in malignant respiratory diseases. This technology has the potential to greatly improve disease diagnosis, real-time intraoperative monitoring, and therapeutic efficacy assessment. The integration of CLE with artificial intelligence is indeed an exciting future direction for the development of this technology. This could lead to faster and even more accurate diagnoses, as well as personalized treatment plans. However, as my rightly pointed out, before CLE becomes a standard part of clinical practice, it would be necessary to conduct larger-scale in vivo studies for validation. This will help ensure that the technology is effective and safe for wider use.

## Author contributions

**Conceptualization:** Li Qian, Tang Yilian.

**Data curation:** Tang Yilian.

**Funding acquisition:** Pu Xiang, Chai Yihui.

**Formal analysis:** Fan Zhiliang.

**Methodology:** Fan Zhiliang, Chai Yihui.

**Writing – original draft:** Fan Zhiliang, Li Qian, Tang Yilian, Chai Yihui.

**Writing – review & editing:** Pu Xiang.
